# Costs of First-Line Treatment With FOLFIRINOX, Modified FOLFIRINOX, and Gemcitabine With Nab-Paclitaxel in Metastatic Pancreatic Ductal Adenocarcinoma

**DOI:** 10.36469/001c.142403

**Published:** 2025-08-22

**Authors:** Syvart Dennen, Marty Masek, Paul Cockrum, Elizabeth Nagelhout, Ravi Paluri

**Affiliations:** 1 Genesis Research Group, Hoboken, New Jersey; 2 Ipsen Biopharmaceuticcals Inc., Cambridge, Massachusetts; 3 Atrium Health Wake Forest Baptist Comprehensive Cancer Center, Winston-Salem, North Carolina

**Keywords:** healthcare resource utilization, pancreatic cancer, payer perspectives, supportive care, cost drivers, real-world evidence

## Abstract

**Background:**

Further research is needed to determine real-world costs of first-line (1L) treatment of metastatic pancreatic ductal adenocarcinoma (mPDAC) with FOLFIRINOX (FFX), modified FFX (mFFX), and gemcitabine with nab-paclitaxel (GnP).

**Objectives:**

To describe healthcare costs by treatment regimen, stratified by commercial and Medicare Advantage insurance.

**Methods:**

This retrospective cohort study of adult patients with mPDAC utilized Optum’s de-identified Market Clarity Dataset. Demographics, clinical characteristics, and 1L unadjusted all-cause healthcare costs were examined. Total all-cause costs included costs from inpatient, outpatient, chemotherapy drug and administration, granulocyte colony-stimulating factor (G-CSF), radiation therapy, and other outpatient and pharmacy costs.

**Results:**

A total of 3115 patients met the criteria for inclusion and received 1L treatment with either FFX, mFFX, or GnP. Among those, 1703 had commercial insurance (FFX, 536; mFFX, 673; GnP, 494) and 1412 had Medicare Advantage (FFX, 201; mFFX, 317; GnP, 894). Total cost of care (mean [SD]) was similar between regimens for each insurance cohort (mean [SD] commercial: FFX, 137 813[127 504]; mFFX, 120 109[112 208]; GnP, 133 042[154 248]; Medicare Advantage: FFX, 110 788[98 492]; mFFX, 98 667[83 437]; GnP, 110 211[100 150]). For both insurance cohorts, chemotherapy drug costs were highest for GnP (mean [SD] commercial: FFX, 10 916[21 647]; mFFX, 7653[10 054]; GnP, 60 466[112 589]; Medicare Advantage: FFX, 8028[11 044]; mFFX, 6016[7688]; GnP, 49 263[49 373]), while chemotherapy administration costs were higher for FFX and mFFX (commercial: FFX, 25 458[33 350]; mFFX, 22 795[24 309]; GnP 12 206[15 766]; Medicare Advantage: FFX, 25 512[36 352]; mFFX, 21 524[22 317]; GnP 11 103[13 089]). G-CSF costs were also higher for FFX and mFFX (commercial: FFX, 38 074[56 593], mFFX, 27 823[41 166]; GnP, 4029[14 181]; Medicare Advantage: FFX, 30 535[56 630]; mFFX, 24 596[39 286]; GnP, 2412[9115]).

**Discussion:**

Total costs of 1L FFX, mFFX, and GnP were similar within a commercially insured and Medicare Advantage cohort. FFX and mFFX costs were largely driven by chemotherapy administration and G-CSF costs, while GnP costs were driven by chemotherapy drug costs.

**Conclusions:**

To fully assess the economic impact of mPDAC in 1L treatment, it is essential to consider both the total cost and the individual cost components, such as chemotherapy drugs, administration, and supportive care costs.

## BACKGROUND

Pancreatic cancer remains one of the deadliest malignancies, behind only lung and colon cancer in mortality rates.^1,2^ Pancreatic ductal adenocarcinoma (PDAC) is the most common type of pancreatic cancer, occurring in approximately 90% of cases.^3^ Early symptoms are often absent, which frequently contributes to delayed diagnosis.^3^ Combined with the aggressive nature of this type of cancer, less than 20% of patients have resectable disease when they are diagnosed.^4^ Additionally, approximately 50% of patients have distant metastases at diagnosis,^1^ further contributing to the poor survival rates in this disease. The 5-year relative survival is only 3.1% in patients with distant metastases.^1^

The costs of pancreatic cancer are also high, largely due to the frequency of cases of advanced disease at diagnosis.^5^ Among gastrointestinal cancers, pancreatic cancer has the second-highest systemic costs, following colorectal cancer.^5^

In patients with metastatic PDAC (mPDAC) and Eastern Cooperative Oncology Group Performance Status (ECOG PS) scores of 0-1, NCCN Category 1 Preferred first-line (1L) treatment options within NCCN Clinical Practice Guidelines in Oncology (NCCN Guidelines^®^) include fluorouracil, leucovorin, irinotecan, and oxaliplatin (FOLFIRINOX [FFX]), liposomal irinotecan, oxaliplatin, leucovorin, and fluorouracil (NALIRIFOX), and gemcitabine with nab-paclitaxel (GnP).^6^ Modified FFX (mFFX) is also included as a Category 2A preferred treatment option within NCCN Guidelines^®^ in these patients.^6^

Both FFX and GnP are associated with significant toxicity.^7,8^ FFX has been modified in clinical practice through dose reductions or omission of regimen components, such as the 5-fluorouracil (5FU) bolus, to increase tolerability.^9^ Similarly, intervals between administrations of GnP can be modified, such as omitting the week 2 dose.^10,11^ On February 13, 2024, irinotecan liposome with oxaliplatin, fluorouracil, and leucovorin was approved by the US Food and Drug Administration (FDA) for the 1L treatment of mPDAC.^12^ The NAPOLI 3 phase III clinical trial found a significantly longer median overall survival for NALIRIFOX compared with GnP (11.1 months [95% CI, 10.0-12.1] vs 9.2 months [95% CI, 8.3-10.6]).^13^

Currently, FFX, modified FFX (mFFX), and GnP are the most widely used 1L treatment options, and further research is needed to understand the impact of their real-world costs on the health system. We performed a retrospective analysis of claims data from real-world patients with mPDAC receiving each of these regimens. We calculated total all-cause healthcare costs incurred by patients being treated for mPDAC. Total costs and their various components were stratified by 1L treatment (FFX, mFFX, and GnP) and primary payer (commercial insurance and Medicare Advantage). In addition, we describe costs of branded and generic nab-paclitaxel (nP) among 1L GnP patients, and report branded costs in the full study period as well as post-generic entry. To our knowledge, no previous studies have assessed real-world costs associated with generic vs branded nP and whether the entry of generic nP impacted costs.

Due to the recent approval of NALIRIFOX at the time this study was undertaken, sufficient data were not yet available to include it in the analysis. Future research is needed to assess its real-world outcomes.

## METHODS

### Study Design and Data Source

This noninterventional retrospective cohort study utilized the Optum® Market Clarity Dataset. It is composed of medical and pharmacy claims linked with electronic health record (EHR) data from providers across the continuum of care. The data are de-identified and cannot be used to determine the identity of the subjects. Based on US federal regulations (45 CRF 46.104 – Exempt Research), this study is exempt from institutional review board review.

### Study Population and Participants

Patients at least 18 years old with a diagnosis of mPDAC from January 1, 2015, through May 31, 2023, were included in the study. Diagnosis of mPDAC was defined by both a pancreatic cancer diagnosis and a metastatic diagnosis. The pancreatic cancer diagnosis was determined by the presence of at least 1 inpatient or at least 2 outpatient claims with diagnosis codes ICD-9-CM 157.xx (excluding 157.4) or ICD-10-CM C25.xx (excluding C25.4) at least 30 days and up to 1 year apart. The metastatic diagnosis date was the earliest of at least 1 inpatient or at least 2 outpatient claims with diagnoses for secondary malignancies (ICD-9-CM: 196.xx-198.xx, ICD-10-CM: C77.xx-C79.xx) separated by at least 30 days and up to 1 year. The metastatic diagnosis was required to be at least 30 days prior to or up to 1 year after the pancreatic cancer diagnosis. Other inclusion criteria were initiation of 1L FFX, mFFX, or GnP up to 14 days before and up to 90 days after metastatic diagnosis, with treatment start defined as index date; at least 6 months of continuous medical enrollment pre-index (baseline period) and at least 30 days of continuous medical and pharmacy enrollment post-index, unless death occurred first. Death dates were set to the last day of the month.

Exclusion criteria were enrollment in a clinical trial, determined by the presence of ICD-9-CM V70.7 or ICD-10-CM Z00.6; a diagnosis of other primary or secondary malignancies (excluding non-melanoma skin cancer) up to 6 months and at least 30 days prior to pancreatic cancer diagnosis, based on at least 1 inpatient or at least 2 outpatient claims at least 30 days apart; treatment with systemic pancreatic cancer therapies (listed in **Supplementary Table S1**) ≥15 days before index; a death date prior to index.

Full details on 1L therapy definitions are available in **Supplementary Table S1**. Briefly, the start of 1L therapy was the first administration or fill of an eligible therapy up to 14 days before and up to 90 days after the metastatic diagnosis. Regimens were defined as the combination of all eligible therapies administered in the first 28 days of treatment. A new eligible treatment administered more than 28 days after line initiation began a new line of therapy (LOT), with 1L ending at the earliest of either (1) the day before the new LOT began or (2) final 1L drug administration + 28 days. A treatment gap of at least 90 days ended the LOT at the date of last 1L drug administration + 28 days. In the absence of a treatment gap or initiation of a new LOT, the end date of 1L was the earliest of either (1) the death date or (2) the date of last 1L drug administration + 28 days. Patients were excluded from the analysis if their LOT was considered incomplete due to censoring by end of continuous claims enrollment during treatment, defined as disenrollment without death less than 28 days after last observed 1L drug administration.

FFX was defined as the regimen including 5FU, irinotecan, oxaliplatin, and leucovorin, with 5FU administered as both a bolus and infusion in the first cycle. Modified FFX was defined as FFX without 5FU bolus in the first cycle. GnP was defined as the regimen containing gemcitabine with nP. Additional information on regimens definitions can be found in the **Supplementary Material.**

### Outcomes

Demographics and clinical characteristics were assessed during the baseline period and reported by insurance type (commercial or Medicare Advantage) and regimen.

For all 3 treatment groups (FFX, mFFX, and GnP), 1L total unadjusted all-cause healthcare costs (TCoC) were calculated and reported by insurance type (commercial or Medicare Advantage). TCoC was divided into the mutually exclusive categories of inpatient costs (ICU- and non-ICU-related) and total outpatient costs. The latter were further subdivided into costs for chemotherapy drugs; chemotherapy administration (injection/infusion costs and non-G-CSF supportive care, such as antiemetics, hydration, and erythropoiesis-stimulating agents); G-CSF acquisition and administration; radiation therapy; other outpatient medical (costs under the medical benefit not included in the previous components, such as office visits, labs, and physician-administered drugs not included in the other categories); and other outpatient pharmacy (costs under the pharmacy benefit).

Costs in the database were available in the form of a standardized cost variable created by Optum through a multistep proprietary algorithm. The process removes differences in pricing across health plans, provider contracts, and the associated regional variation in payments; Optum states that the resulting costs can be viewed as if they were derived from a single source with a single approach to classifying and pricing services. Standardized cost is an estimate of the allowed amount, which includes both payer and patient liabilities, and is therefore an estimate of total expenditure across both parties. Please see the **Supplementary Material** for further information on the methodology employed to create this variable.

To further understand drivers of cost differences, mean chemotherapy administration costs were examined in a subgroup of patients with linked EHR data during 1L. These costs are stratified by CPT and Healthcare Common Procedure Coding System (HCPCS) codes for procedures and medications, such as infusion/injection of regimen drugs and supportive care (except G-CSF), such as antiemetics, hydration infusions, and pain medication.

The analysis of branded vs generic nP was performed at the claim level and the patient level. At the claim level, nP claims were divided into generic vs branded based on National Drug Codes (NDC) on the claim. Claims with missing NDC were excluded.

At the patient level, GnP patients were divided into branded vs generic groups: the first with only administrations of branded nP (Abraxane), the second with only administrations of generic nP. Patients were required to have at least 30 days of follow-up in 1L, no nP claims missing NDCs, and no mixed branded and generic nP administrations.

Branded and generic nP costs were assessed from April 1, 2022, to the end of the study period. To assess the impact of generic entry, branded nP cost was also calculated during the full study period.

### Statistical Analysis

All results are presented as descriptive statistics, with count and percentage for demographics and clinical characteristics and mean (SD), median (interquartile range [IQR]), and range for costs.

For mean TCoC, 1-way Welch’s ANOVA was performed for all 6 regimen-payer groups, followed by a Games-Howell test for pairwise differences. For cost categories, 1-way Welch’s ANOVA was performed across the 3 regimens separately, stratified by payer type. If the ANOVA was statistically significant, a 2-sided Games-Howell test was performed to assess pairwise differences across the 3 regimens. The significance level for both tests was prespecified as ɑ = 0.05. While the Games-Howell test adjusts for multiple comparisons, we did not additionally adjust for multiple comparisons across all tests performed. Results should be viewed as descriptive; nominal *P* values are reported to provide context for observed effect sizes relative to variability.

Welch’s ANOVA and the Games-Howell tests are robust to non-normality with increasing sample size.^14^ Previous research on healthcare costs has found that sample sizes of 200, and less in some cases, are sufficient for standard statistical tests to be accurate.^15,16^ Sample sizes in our study ranged from 201 to 894 patients.

We employed compact letter display (CLD) to show statistically significant differences between groups in **Figures 2, 3, and 4**. Groups with no evidence of a difference share a letter, while groups that are statistically significantly different have no common letters. *P* values and 95% CIs for all pairwise tests are available in **Supplementary Table S4**.

Costs for the branded vs generic nP groups were adjusted to PPPM by dividing the sum of each patient’s costs by the duration of therapy in months. This controlled for the variation in treatment duration in the small sample of patients meeting inclusion criteria for this analysis.

Costs were adjusted for inflation to 2023 US dollar values (USD) using the medical care components of the Consumer Price Index. All data analyses were carried out using R 4.3.1.

## RESULTS

### Participants

**Figure 1.**
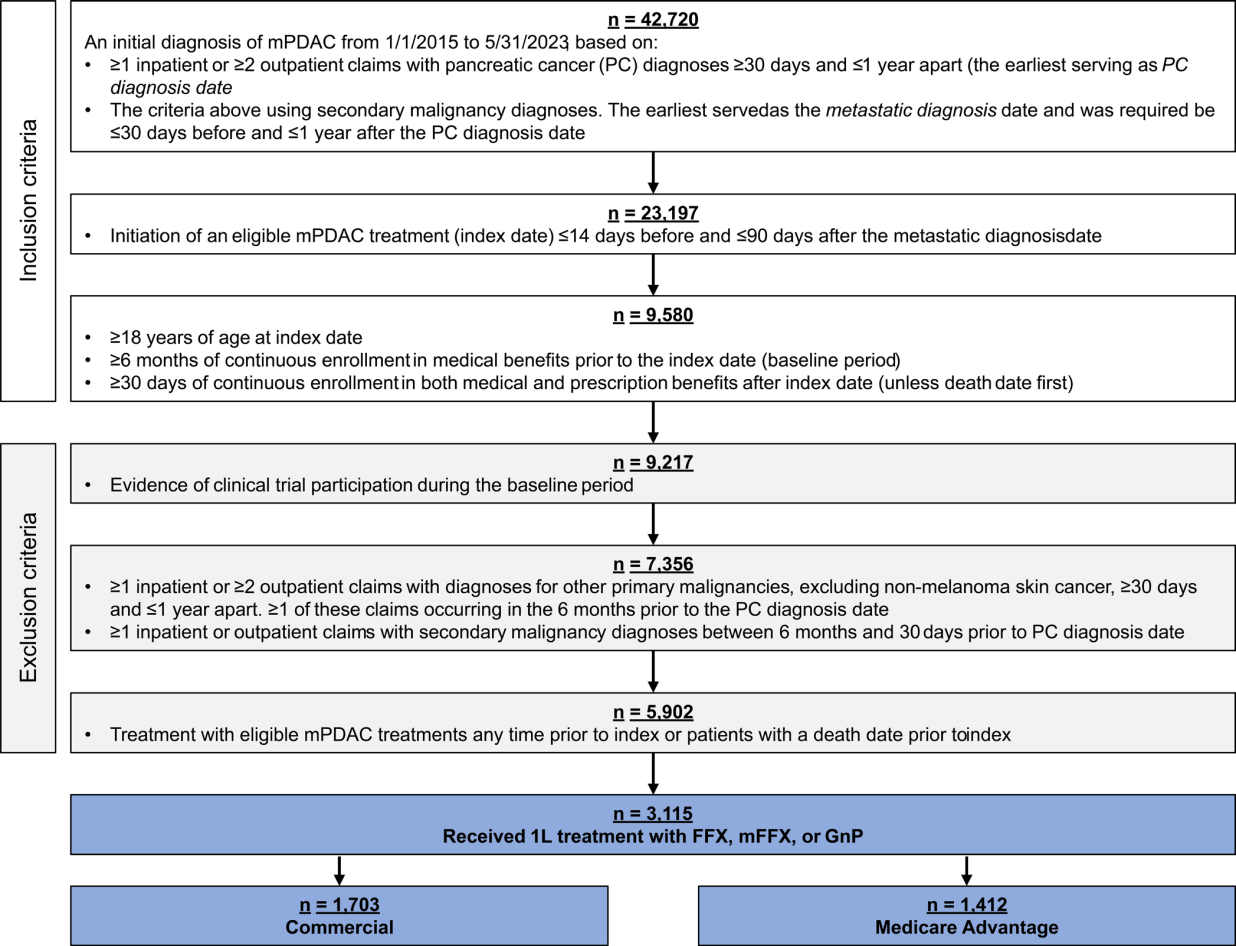
Cohort Attrition Abbreviations: FFX, FOLFIRINOX (5-fluorouracil, leucovorin, irinotecan, and oxaliplatin); GnP, gemcitabine with nab-paclitaxel; mFFX, modified FOLFIRINOX; mPDAC, metastatic pancreatic ductal adenocarcinoma.

There were 42 720 patients with a diagnosis of mPDAC meeting the initial inclusion criteria (**Figure 1**). The final analytic cohort was comprised of 3115 patients that met the remaining inclusion/exclusion criteria and received 1L treatment with either FFX, mFFX, or GnP. Stratifying by payer, 1703 of these patients had commercial insurance (55%) and 1412 had Medicare Advantage (45%). In the commercial group, 536 patients received FFX (31%), 673 mFFX (40%), and 494 GnP (29%). In the Medicare Advantage group, 201 patients received FFX (14%), 317 mFFX (22%), and 894 GnP (63%).

### Baseline Demographics and Patient Characteristics

Baseline patient demographics and clinical characteristics for each regimen and insurance type are shown in **Table 1**. Among patients with commercial insurance, mean age was higher in the 1L GnP group than the 1L FFX and mFFX groups (GnP, 62; FFX, 58; mFFX, 58 years). Patients with Medicare Advantage had a greater mean age than commercial patients for each treatment. As in the commercial group, GnP patients had greater mean age than those treated with FFX and mFFX (GnP, 74; FFX, 70; mFFX, 71 years). In the commercial group, FFX patients were notably more male (63% male vs 37% female). This difference was less pronounced for mFFX and GnP (mFFX, 55% male vs 45% female or unknown; GnP, 55% male vs 45% female). In the Medicare Advantage group, the sex distribution was more evenly split, with male vs female: FFX, 55% vs 45%; mFFX, 50% vs 50%; GnP, 48% vs 52%. Most patients were White across all insurance types and treatment regimens (≥68%).

**Table 1.** Demographics Among Commercial and Medicare Advantage Insurance Patient Groups Treated With 1L FFX, mFFX, or GnP

	**Commercial**			**Medicare Advantage**		
**1L FFX**	**1L mFFX**	**1L GnP**	**1L FFX**	**1L mFFX**	**1L GnP**	
Total patients, n	536	673	494	201	317	894
Age at index date						
Mean (SD)	58 (8)	58 (8)	62 (8)	70 (7)	71 (6)	74 (6)
Median (IQR)	59 (53-63)	59 (54-63)	62 (58-66)	70 (67-74)	71 (68-74)	74 (70-78)
Range	32, 80	29, 83	32, 88	39, 85	41, 86	50, 88
Age (y) at index date, n (%)						
18-35	NR	5 (0.7)	NR	0 (0)	0 (0)	0 (0)
36-50	86 (16)	84 (12)	NR	NR	NR	NR
51-64	362 (68)	473 (70)	306 (62)	NR	NR	NR
65-74	81 (15)	99 (15)	115 (23)	125 (62)	221 (70)	421 (47)
≥75	NR	12 (1.8)	42 (8.5)	45 (22)	71 (22)	425 (48)
Sex, n (%)						
Female or unknown^a^	198 (37)	300 (45)	220 (45)	90 (45)	159 (50)	463 (52)
Male	338 (63)	373 (55)	274 (55)	111 (55)	158 (50)	431 (48)
Race, n (%)						
Asian	8 (1.5)	10 (1.5)	10 (2.0)	5 (2.5)	5 (1.6)	13 (1.5)
Black or African American	37 (6.9)	38 (5.6)	40 (8.1)	17 (8.5)	34 (11)	102 (11)
Other/unknown^a^	111 (21)	127 (19)	105 (21)	34 (17)	62 (20)	157 (18)
White or Caucasian	380 (71)	498 (74)	339 (69)	145 (72)	216 (68)	622 (70)
Year of index, n (%)						
2014-2018	252 (47)	145 (22)	256 (52)	75 (37)	58 (18)	359 (40)
2019-2023	284 (53)	528 (78)	238 (48)	126 (63)	259 (82)	535 (60)
Region, n (%)						
Northeast	160 (30)	136 (20)	84 (17)	63 (31)	83 (26)	191 (21)
Midwest	214 (40)	274 (41)	189 (38)	92 (46)	139 (44)	400 (45)
South	112 (21)	173 (26)	150 (30)	34 (17)	56 (18)	195 (22)
West	34 (6.3)	65 (9.7)	54 (11)	7 (3.5)	24 (7.6)	84 (9.4)
Other/unknown^a^	16 (3.0)	25 (3.7)	17 (3.4)	5 (2.5)	15 (4.7)	24 (2.7)

Abbreviations: 1L, first line; FFX, FOLFIRINOX (fluorouracil, leucovorin, irinotecan, and oxaliplatin); GnP, gemcitabine with nab-paclitaxel; mFFX, modified FOLFIRINOX; NR, not reported.

Because of rounding, percentages may not total 100. Optum guidelines prohibit reporting cells of <5 patients, including reporting cell values that allow a count of <5 to be derived. Unknown was combined with the next smallest category, as applicable, due to this rule.

Baseline patient comorbidities are shown in **Supplementary Table S2**. Common baseline comorbidities included hypertension, diabetes, cardiac arrhythmias, peripheral vascular disorders, obesity, COPD, and depression.

Among patients with commercial insurance, FFX patients tended to have lower proportions of baseline comorbidities than mFFX and GnP patients, suggesting that FFX patients were slightly healthier than mFFX and GnP patients. The proportion of FFX, mFFX, and GnP patients with uncomplicated hypertension was, respectively, 53%, 58%, and 67%; cardiac arrhythmia: 17%, 21%, and 23%; uncomplicated diabetes: 31%, 34%, and 41%; COPD: 15%, 15%, and 22%.

Among patients with Medicare Advantage, a similar pattern was present. The proportion of FFX, mFFX, and GnP patients with uncomplicated hypertension was, respectively, 76%, 77%, and 79%; cardiac arrhythmias: 25%, 26%, and 33%; uncomplicated diabetes: 44%, 44%, and 46%; COPD: 21%, 24%, and 28%.

In line with their greater age, the Medicare Advantage group tended to have more comorbidities than the commercial group, particularly for hypertension and peripheral vascular disorders. In contrast, proportions of patients with liver disease were slightly higher in the commercial group vs the Medicare Advantage group.

### Healthcare Costs

Among patients with commercial insurance, costs were calculated over a median (IQR) treatment duration, in months, of: FFX, 4.6 (2.3-7.4); mFFX, 4.6 (2.1-6.9); GnP, 3.7 (2.1-6.2). Among patients with Medicare Advantage, the median (IQR) durations were: FFX, 3.9 (2.1-7.2); mFFX, 3.5 (1.9-6.2); GnP, 3.3 (1.9-6.0).

### Total Cost of Care

This section presents the mean, SD, and *P* values for difference of means. For median, IQR, and range, please see **Supplementary Table S3**. For full ANOVA *P* values and Games-Howell 95% CIs and *P* values, please see **Supplementary Table S4**.

**Figure 2.**
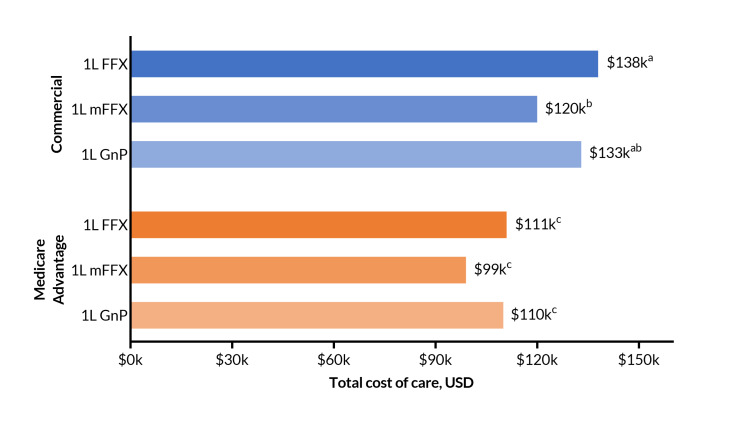
Total Costs Among Commercial and Medicare Insurance Groups Treated With 1L FFX, mFFX, or GnP Abbreviations: FFX, FOLFIRINOX (5-fluorouracil, leucovorin, irinotecan, and oxaliplatin); GnP, gemcitabine with nab-paclitaxel; mFFX, modified FOLFIRINOX; USD, US dollars. Compact letter display (CLD) is used to show statistically significant differences. Categories with a letter in common show no evidence of a difference, while categories without a letter in common show a statistically significant difference at ɑ = 05. Full results of the ANOVA and Games-Howell tests are available in **Supplementary Table S4.**

The results of the ANOVA assessing mean TCoC across all 6 groups (FFX, mFFX, and GnP, both commercial and Medicare), indicated statistically significant differences for one or more groups (*P* < .001). Among patients with commercial insurance, TCoC was similar in magnitude between treatment groups (**Figure 2**), with mean (SD) TCoC of: FFX, $137 813 ($127 504); mFFX, $120 109 ($112 208); GnP $133 042 ($154 248). While mFFX had a statistically significant 13% lower cost than FFX (*P* = .005), mFFX and GnP were not significantly different (*P* = .222).

The pattern was similar among patients with Medicare Advantage: mean (SD) TCoC was FFX, $110 788 ($98 492); mFFX, $98 667 ($83 437); GnP, $110 211 ($100 150) (**Figure 2**). Differences in these means were not statistically significant (*P* >.05 for all pairwise comparisons). Across payers, mean TCoC for each regimen was approximately 20% higher for commercial vs Medicare Advantage (*P* < .001 for the 3 pairwise comparisons of each regimen across payer type).

### Inpatient Costs

**Figure 3.**
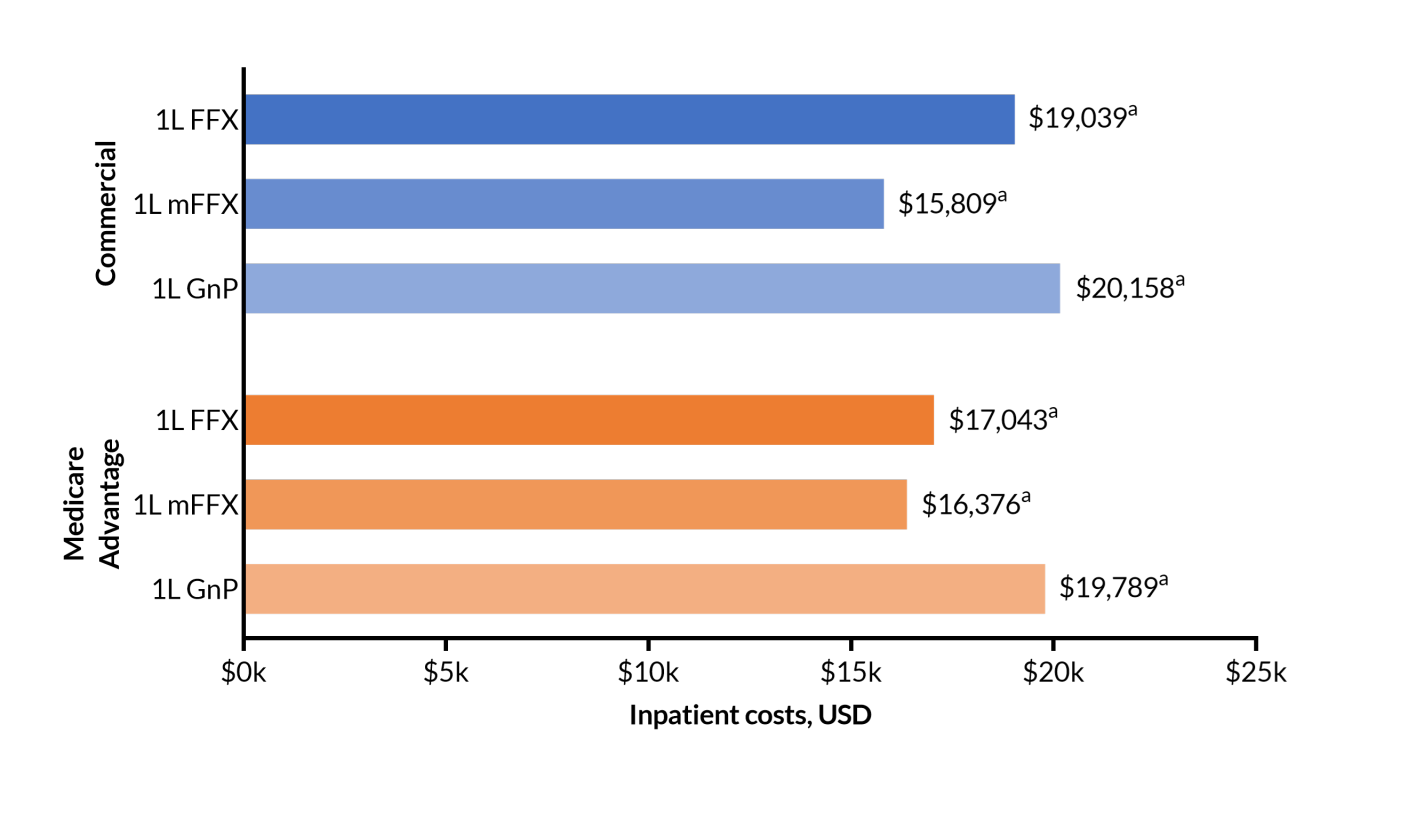
Inpatient Costs Among Commercial and Medicare Insurance Groups Treated With 1L FFX, mFFX, or GnP Abbreviations: FFX, FOLFIRINOX (5-fluorouracil, leucovorin, irinotecan, and oxaliplatin); GnP, gemcitabine with nab-paclitaxel; mFFX, modified FOLFIRINOX; USD, US dollars. See **Figure 2** footnote or the methods section for an explanation of the lettering system for statistically significant differences. Testing is within payer type; Medicare and commercial cost were not calculated for this outcome.

Among patients with commercial insurance, mean (SD) all-cause inpatient costs were: FFX, $19 039 ($35 471); mFFX, $15 809 ($33 733); GnP, $20 158 ($42 729). Although mFFX had somewhat lower cost, there was no statistically significant difference across the regimens (*P* = .106). For the Medicare Advantage group, differences in means were also not statistically significantly different (*P* = .295). Mean (SD) inpatient costs were: FFX, $17 043 ($29 173); mFFX, $16 376 ($31 804); GnP, $19 789 ($45 094) (**Figure 3**). Non-ICU costs were the predominant component of inpatient costs.

### Outpatient Costs

**Figure 4.**
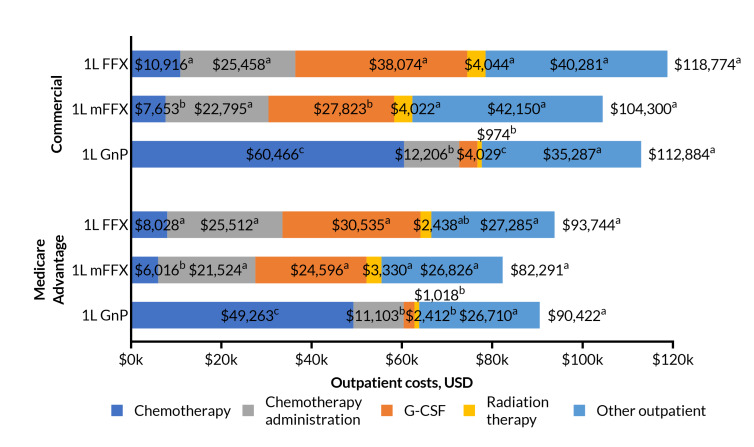
Outpatient Costs Among Commercial and Medicare Insurance Groups Treated With 1L FFX, mFFX, or GnP Abbreviations: FFX, FOLFIRINOX (5-fluorouracil, leucovorin, irinotecan, and oxaliplatin); GnP, gemcitabine with nab-paclitaxel; mFFX, modified FOLFIRINOX; USD, US dollars. See Figure 2 footnote or the methods section for an explanation of the CLD lettering system for statistically significant differences. Testing is within payer type; Medicare and commercial cost were not calculated for these outcomes. A small number of patients were excluded from subcategories where their cost was negative. Due to this, the sum of the subcategories may differ by a small amount from the total outpatient cost for some treatment regimens and insurance types. The other outpatient costs subcategory in this figure includes both other outpatient medical and other outpatient pharmacy, reported separately in the **Supplementary Material.**

Among patients with commercial insurance, mean all-cause outpatient costs were also similar between groups, though slightly lower for mFFX. The mean (SD) was: FFX, $118 774 ($121 265); mFFX, $104 300 ($104 318); GnP, $112 884 ($142 080). Differences in means were not statistically significant (*P* = .082). A similar pattern was present in Medicare Advantage, with mean (SD) outpatient costs: FFX, $93 744 ($95 444); mFFX, $82 291 ($76 170); GnP, $90 422 ($81 371). Differences were not statistically significant (*P* = .203). (**Figure 4**).

Chemotherapy drug and administration costs, both components of outpatient costs, differed between the three regimens within each payer type. Drug costs were much larger in magnitude for GnP relative to FFX and mFFX, while administration costs for FFX and mFFX substantially exceeded those of GnP.

For commercially insured patients, mean (SD) chemotherapy drug costs were FFX, $10 916 ($21 647); mFFX $7653 ($10 054); GnP, $60 466 ($112 589). Pairwise tests of differences were all statistically significant (*P* <. 001), although the observed difference between FFX and mFFX was small relative to GnP. The Medicare Advantage group followed a similar pattern, with the following mean (SD) drug costs: FFX, $8028 ($11 044); mFFX, $6016 ($7688); GnP, $49 263 ($49 373) (*P* < .001 for FFX and mFFX vs GnP; *P* = .004 for FFX vs mFFX) (**Figure 4**).

In contrast, chemotherapy administration costs for FFX and mFFX were similar and statistically significantly higher than for GnP. For commercial patients, mean (SD) costs were FFX, $25 458 ($33 350); mFFX, $22 795 ($24 309); GnP, $12 206 ($15 766) (*P* < .001 for FFX and mFFX vs GnP; *P* = .073 for FFX vs mFFX). For Medicare Advantage patients, the costs were FFX, $25 512 ($36 352); mFFX, $21 524 ($22 317); GnP, $11 103 ($13 089) (*P* < .001 for FFX and mFFX vs GnP; *P* = .120 for FFX vs mFFX) (**Figure 4**).

G-CSF costs were highest for FFX, slightly lower for mFFX, and lowest for GnP. For commercial patients, the mean (SD) was: FFX, $38 074 ($56 593); mFFX, $27 823 ($41 166); GnP, $4029 ($14 181). All differences were statistically significant at *P* < .001. For Medicare Advantage patients, mean (SD) G-CSF costs were: FFX, $30 535 ($56 630); mFFX, $24 596 ($39 286); GnP, $2412 ($9115). FFX and mFFX vs GnP were statistically significant (*P* < .001), while FFX vs mFFX was not (*P* = .158) (**Figure 4**).

Among commercial patients, mean (SD) radiation therapy costs were similar for FFX and mFFX, at $4044 ($18 314) and $4022 ($17 477), while GnP was lower at $974 ($8165) (*P* < .001 for FFX and mFFX vs GnP; *P* > .999 FFX vs mFFX). In the Medicare Advantage group, mean (SD) costs were FFX, $2438 ($12 016); mFFX, $3330 ($13 893); GnP, $1018 ($7548). Only mFFX and GnP were statistically significantly different (*P* < .001 for mFFX vs GnP; *P* = .518 for FFX vs mFFX; *P* = .062 for FFX vs GnP) (**Figure 4**).

### Chemotherapy Administration Cost Components

In a subgroup of patients of any payer type, the additional cost of chemotherapy administration for FFX and mFFX vs GnP primarily came from claim lines with HCPCS and CPT codes related to FFX and mFFX’s greater duration of administration and number of component drugs, as well as costs for antiemetic medications. Claim lines with CPT codes related to the 5FU infusion pump had mean costs that were $2115 and $2101 higher for FFX and mFFX vs GnP, while lines with a CPT code for additional hours of infusion were, on average, $1250 and $1497 higher for FFX and mFFX vs GnP, respectively. Mean costs from a HCPCS code for fosaprepitant injection were $1125 and $1139 higher for FFX and mFFX vs GnP, while a code for aprepitant injection was $906 and $1135 higher, respectively. Other codes that had higher costs for FFX and mFFX vs GnP were related to additional infusions/injections, the 5FU pump, or supportive medications. A table of mean costs and differences by treatment group for HCPCS/CPT codes related to administration is provided in **Supplementary Table S5** for all codes with a difference in means of >$100 between FFX or mFFX vs GnP.

### Generic vs Branded Costs of nP

There were 308 generic and 343 branded claims for nP during the period of generic competition (April 2022–May 2023). The mean generic nP cost was affected by outliers, as seen by the large SD and range, and was more than double the branded mean (**Table 2**). This difference is much less pronounced when considering the median (IQR), which is less affected by outliers. On generic claims these were $5890 ($3482-$6964) vs $3482 ($2945-$6583) for branded. Expanding to the full study period, the median (IQR) for branded nP was nearly identical, at $3558 ($2945-$6583), showing no discernible impact of generic entry.

**Table 2.** Costs per nP Claim and All-Cause Healthcare Costs for Generic vs Branded nP

	**Generic Competition Period (April 2022–May 2023)**		**Full Study Period (Jan. 2015–May 2023)**
	**Generic nP**	**Branded nP**	**Branded nP**
Claim-level results			
Total claims, n	308	343	5054
Mean (SD)	11 761 (40 559)	4721 (2 197)	5451 (13 582)
Median (IQR)	5890 (3482-6964)	3482 (2945-6583)	3558 (2945-6583)
Range	0-266 890	175-20 893	0-266 750
Patient-level results			
Total patients, n	24	20	305
Total cost of care, USD			
Mean (SD)	52 129 (121 166)	25 410 (9 531)	27 315 (19 550)
Median (IQR)	24 313 (17 231-36 774)	22 276 (17 911-32 180)	21 625 (16 279-31 981)
Range	9285-616 941	11 816-40 501	5637-157 571
Inpatient cost, USD			
Mean (SD)	7162 (11 153)	5554 (8 624)	7052 (16 035)
Median (IQR)	0 (0-13 735)	0 (0-8151)	0 (0 6983)
Range	0-36 048	0-26 901	0-123 785
Outpatient cost, USD			
Mean (SD)	44 967 (118 241)	19 856 (7456)	20 263 (10 124)
Median (IQR)	20 115 (16 396-26 663)	18 291 (14 490-23 581)	18 429 (14 488-23 625)
Range	9285-599 243	9673-40 501	4314-112 991
Chemotherapy drug cost, USD			
Mean (SD)	35 910 (118 205)	11 056 (4334)	10 497 (5930)
Median (IQR)	10 554 (7162-16 797)	10 162 (7658-13 508)	9581 (6976-13 450)
Range	5431-590 252	5947-23 508	738-76 410

Abbreviations: PPPM, per-patient-per-month; nP, nab-paclitaxel; USD, US dollars.

The patient-level analysis was similarly affected by outliers. Sample sizes were small, with 24 patients in the generic group and 20 in the branded group. During the period of generic competition, the generic group mean PPPM TCoC was approximately twice as high as in the branded group. As in the claims-level analysis, median (IQR) costs were more similar across the groups: in the period of generic competition, median (IQR) PPPM TCoC was $24 313 ($17 231-$36 774) for the generic group and $22 276 ($17 911-$32 180) for the branded group (**Table 2**). The branded group median (IQR) TCoC was almost the same when considering the full study period, at $21 625 ($16 279-$31 981). Examining components of TCoC, mean inpatient costs were broadly similar, while outpatient cost differences in means were driven by chemotherapy drug costs. As with TCoC, median (IQR) PPPM chemotherapy drug costs were nearly the same for the generic and branded groups, at $10 554 ($7162-$16 797) and $10 162 ($7658-$13 508), respectively. As before, the branded group showed little difference when expanded to include the full study period, with a median (IQR) of $9581 ($6976-$13 450).

## DISCUSSION

This real-world study highlights similarities and differences in TCoC and cost drivers in a cohort of patients with mPDAC treated with 1L FFX, mFFX, or GnP. Within each payer type, mean TCoC was similar in magnitude across the 3 regimens, while the categories of costs driving FFX and mFFX vs GnP differed. Chemotherapy administration and G-CSF costs drove overall costs for FFX and mFFX, while chemotherapy drug costs composed a much larger proportion of total GnP costs. The additional cost of chemotherapy administration for FFX and mFFX than with GnP primarily came from the greater number of regimen components clinicians must administer, the longer duration of administration (including 5FU pump), and supportive care costs for antiemetic medications.

Similar studies comparing costs by payer type in this setting are limited. In a real-world study of patients with commercial insurance vs Medicare Fee-For-Service, Tomicki et al^17^ also found similar mean TCoC between 1L FFX and 1L GnP within payer type. The study did not stratify by FFX and mFFX. The authors found mean TCoC was nearly 3 times higher in the commercially insured group vs the Medicare Fee-For-Service group. This contrasts with our study findings, in which the mean TCoC was approximately 1.2 times greater for commercial insurance vs Medicare Advantage. The difference in result is likely due to the standardized cost estimate used in our study, which adjusts prices across health plans and providers to the same payment schedule.

Both our study and Tomicki et al^17^ found that drivers of outpatient costs differed between treatment regimens, with TCoC of FFX (and mFFX in our study) driven by chemotherapy administration and G-CSF costs while TCoC of GnP was driven by chemotherapy drug costs. Similar trends have been noted in other real-world studies.^18–20^

Our study found radiation therapy costs were higher for FFX and mFFX vs GnP, also similar to Tomicki et al.^17^ This may be due to differences in practice patterns associated with treatment selection, or it may indicate a greater need for palliative care among FFX and mFFX patients. A retrospective chart review of mPDAC patients at an academic medical center found radiation therapy applied to the primary tumor reduced reported abdominal pain and the use of opioids.^21^ Palliative radiation therapy for mPDAC is currently being studied in a clinical trial.^22^ Further investigation is warranted to assess the factors underlying this phenomenon and whether it may present opportunities for improved patient management.

We found that mean costs per claim and PPPM were surprisingly higher for generic vs branded nP. Branded nP has long been accepted by payers and clinicians as a component of GnP, despite the previous lack of a generic alternative. During the period of our study, the entry of generic competition had no observable impact on price, leaving the cost equation unchanged. As more real-world data become available, research will be needed to evaluate these findings in a larger sample over a greater time range.

As expected, patients in the commercial insurance group were younger than those in the Medicare Advantage group. Patients among both payer types treated with GnP tended to be older and have more comorbidities than those treated with FFX and mFFX. Due to its toxicity profile, FFX is often selected for younger, fitter patients, while GnP may be better for older patients with comorbidities.^23,24^ The patient characteristics across regimens in our study likely reflect this guidance. However, some studies have found improved overall survival in patients receiving FFX vs GnP across age and ECOG score and fewer hospitalizations following treatment.^25^

Despite the greater age and comorbidities of the GnP group, TCoC was similar across 1L FFX, mFFX, and GnP, with observed variation unlikely to be meaningful from a payer perspective. However, the differences in costs due to chemotherapy administration, G-CSF use, and radiation therapy reflect meaningful differences in clinical practice, patient management, and patient experience during treatment. Future research will be needed to determine the relationship between treatment cost, treatment burden, clinical outcomes, and patient quality of life. In addition, further research is needed to determine the impact of 1L treatment choice on total cost of care over all subsequent treatment.

### Limitations

The objective of this study was to provide a descriptive assessment of costs as observed in the real world. The results should not be used to draw conclusions about direct cost comparisons of one treatment over another. This study did not control for differences in patient characteristics that can drive cost differences, such as comorbidities, age, and socioeconomic conditions. The greatest age and number of comorbidities was observed in the GnP group; adjusting for these factors would likely result in raising cost estimates for FFX and mFFX in relation to GnP.

While Optum’s standardized cost methodology adjusts for variations in price across health plans and provider contracts, our study did not control for differences in care setting and clinical practice, both of which can influence cost. The estimates presented should therefore be viewed as reflecting differences in treatment selection that may be associated with these factors.

We used data through May 31, 2023. Future research will be needed to evaluate more recent trends. The FFX regimen was identified by presence of both a 5FU bolus and infusion in the first administration. This definition ignores the possibility of subsequent dose modification and de-escalation.

## CONCLUSIONS

In this retrospective study of real-world patients with mPDAC, total costs of 1L FFX, mFFX, and GnP were similar within commercially-insured and Medicare Advantage cohorts. FFX and mFFX costs were largely driven by chemotherapy administration and G-CSF, while GnP costs were driven by the component drug price. The higher administration costs for FFX and mFFX were driven by the additional complexity of administering this regimen, including the 5FU infusion pump, and supportive care costs for antinausea/antiemetic medications. The entry of generic nP had no observable impact on the cost of branded nP and the GnP regimen, with branded prices unchanged and the generic price similar or higher.

The results of our study indicate that to fully assess the economic and clinical practice impact of 1L mPDAC treatment, it is essential to consider both TCoC and its drivers. Future research on the relationship between treatment cost, treatment burden, clinical outcomes, and patient quality of life could aid patients and clinicians in selecting the most appropriate treatment.

### Disclosures

S.D., M.M., and E.N. are employees of Genesis Research Group, which received consulting fees from Ipsen Biopharmaceuticals Inc. P.C. reports former employment and equity holder in Ipsen Biopharmaceuticals, Inc. R.P. has received honorarium for consultation and speaking from Ipsen Biopharmaceuticals Inc. and Seagen and for consultation from Exelixis.

## Supplementary Material

Online Supplementary Material

## Data Availability

The data that support the findings of this study are available from Optum, but restrictions apply to the availability of these data, which were used under license for the current study, and so are not publicly available. Data are however available from the authors upon reasonable request and with permission of Optum.
