# Supplementary material for: Costs of First-Line Treatment With FOLFIRINOX, Modified FOLFIRINOX, and Gemcitabine With Nab-Paclitaxel in Metastatic Pancreatic Ductal Adenocarcinoma
Source: J Health Econ Outcomes Res. 2025 Aug 22;12(2):75–84. doi: 10.36469/001c.142403 (PMC12375408; doi:10.36469/001c.142403)
Supplement: Online Supplementary Material [file jheor_2025_12_2_142403_299216.pdf]

Journal of Health Economics and Outcomes Research

Online Supplementary Material

Costs of First-Line Treatment With FOLFIRINOX, Modified FOLFIRINOX, and Gemcitabine With Nab-Paclitaxel in Metastatic Pancreatic Ductal Adenocarcinoma. *JHEOR*. 2025;12(2):75-84. doi:10.36469/jheor.2025.142403

METHODS

Table S1: Line of Therapy Rules and Eligible Systemic Pancreatic Cancer Therapies

Table S2: Baseline Comorbidities Among Commercial and Medicare Advantage Patient Groups Treated With 1L FFX, mFFX, or GnP

Table S3: Healthcare Costs Among Commercial and Medicare Advantage Insurance Patient Groups Treated With 1L FFX, mFFX, or GnP

Table S4: One-Way ANOVA and Games-Howell Test Statistics for Mean Cost Outcomes

Table S5: Chemotherapy Administration Average Cost per HCPCS/CPT Code Among Patients Treated With 1L FFX, mFFX, and GnP

This supplementary material has been provided by the authors to give readers additional information about their work.

This is an open-access article distributed under the terms of the Creative Commons Attribution 4.0 International License (CCBY-4.0). View this license's legal deed at http://creativecommons.org/licenses/by/4.0 and legal code at http://creativecommons.org/licenses/by/4.0/legalcode for more information.

METHODS

FOLFIRINOX Definition

Patients were categorized as having received FOLFIRINOX (FFX) if they had both bolus and infusion during the first cycle (first 14 days of treatment). If on any of these days there was a claim for 5-fluorouracil (5-FU; HCPCS J9190) with either of the 2 CPT codes below, the patient was considered to be in the FFX category:

- 96409: Chemotherapy administration; intravenous, push technique, single or initial substance/drug
- 96411: Chemotherapy administration; intravenous, push technique, each additional substance/drug (List separately in addition to code for primary procedure)

Modified FOLFIRINOX Definition

5-FU not administered as a bolus was determined by the absence of either of the above CPT codes for bolus administration on all days of the first cycle containing claims for 5-FU (based on HCPCS J9190).

National Drug Codes Used for Branded vs Generic Nab-Paclitaxel Analysis

The National Drug Code (NDC) used for branded nab-paclitaxel (nP) was 68817013450. NDC codes used for generic nP were 24979071051, 60505623004, and 00517430001.

Optum Standardized Cost Variable Methodology

Optum applies pricing algorithms to create standardized estimates of the allowed amount across all provider services. The approach varies by service category.

For inpatient stays, the cost is based on MS-DRG when present, otherwise a per-diem cost is applied. The per-diem cost is based on a model of >200,000 inpatient stays accounting for discharge diagnosis, presence of surgery, SNF admission, and length of stay.

Outpatient facility costs are based on the framework of the CMS Outpatient Prospective Payment System.

Professional and ancillary services use a resource-based relative value scale. This allows a standard cost to be estimated across professional services performed by different physician specialties and other clinicians. The relative value unit assigned to a service in Medicare is adjusted upward by a conversion factor to bring costs up to commercial/Medicare Advantage pricing levels.

Pharmacy pricing is created by taking prices available from First Databank and adjusted by therapeutic category and generic indicator. The adjusted prices are then modified based on an Optum analysis of actual allowed amounts on claims to match the real-world pricing level. This creates a payment schedule that is applied to pharmacy claims based on listed NDC and metric quantity.

Table S1. Line-of-Therapy Rules and Eligible Systemic Pancreatic Cancer Therapies

Definition of 1L	• Lines of therapy (LOT) were defined as the combination of all eligible drugs given within 28 days of line start. First line (1L) start was the date of first administration of any eligible therapy (see below for list of eligible therapies).<br>• For GnP only, 1L included the addition of protein-bound paclitaxel to a gemcitabine regimen, or vice versa, within 90 days of line start.
1L start date	• The start of 1L initiation was the earliest date any eligible therapy was given after, or ≤14 days before, the date of metastatic diagnosis.<br>• Eligible therapies and dates were included from claims sources only and not from electronic health records.
1L end date	• Treatment gaps were measured starting the day after an administration ended. That administration date +28 days became the line end date if a gap of ≥90 days followed, with any eligible drug after the gap initiating a new line.<br>• The administration or prescription of any new eligible drug >28 days after 1L start began 2L, with exceptions described below. In this case, the line end date was the lesser of last 1L administration + 28 days or the day before 2L began.<br>• For patients who died without 2L, line end date was the earliest of last administration in 1L + 28 days or death date.<br>The following additions or changes in therapies did not affect 1L end or subsequent line start.<br>• Substitution of 5FU for capecitabine or vice versa.<br>• Substitution of reference products for biosimilars and vice versa<br>• Addition of leucovorin or levoleucovorin to a regimen, or substitution of leucovorin for levoleucovorin, or vice versa, at any time during a LOT. However, presence of leucovorin/levoleucovorin was required within the first 28 days of treatment for a regimen to be considered FFX/mFF
Eligible systemic pancreatic cancer therapies	The therapies below were eligible to initiate and be included as part of a regimen.<br>• Adagrasib<br>• Atezolizumab<br>• Bevacizumab<br>• Capecitabine<br>• Carboplatin<br>• Cisplatin<br>• Dabrafenib<br>• Docetaxel<br>• Dostarlimab<br>• Entrectinib<br>• Erlotinib<br>• Fluorouracil<br>• Fluoropyrimidine<br>• Gemcitabine<br>• Ipilimumab<br>• Irinotecan<br>• Irinotecan liposomal<br>• Larotrectinib<br>• Niraparib<br>• Nivolumab<br>• Olaparib<br>• Oxaliplatin<br>• Paclitaxel<br>• Pembrolizumab<br>• Rucaparib<br>• Selpercatinib<br>• Sotorasib<br>• Trametinib

Abbreviations: 1L, first line; LOT, line of therapy.

Table S2. Baseline Comorbidities Among Commercial and Medicare Advantage Patient Groups Treated With 1L FFX, mFFX, or GnP

	**Commercial**			**Medicare Advantage**		
	**1L FFX**	**1L mFFX**	**1L GnP**	**1L FFX**	**1L mFFX**	**1L GnP**
Total patients, n	536	673	494	201	317	894
Comorbidity, n (%)						
Congestive heart failure	17 (3)	27 (4)	34 (7)	14 (7)	30 (10)	115 (13)
Cardiac arrhythmias	92 (17)	142 (21)	116 (24)	50 (25)	81 (26)	298 (33)
Valvular disease	37 (7)	41 (6)	49 (10)	23 (11)	37 (12)	137 (15)
Pulmonary circulation disorders	32 (6)	47 (7)	54 (11)	18 (9)	28 (9)	87 (10)
Peripheral vascular disorders	50 (9)	80 (12)	75 (15)	40 (20)	77 (24)	209 (23)
Hypertension, uncomplicated	283 (53)	393 (58)	332 (67)	153 (76)	244 (77)	708 (79)
Hypertension, complicated	20 (4)	45 (7)	57 (12)	30 (15)	55 (17)	183 (21)
Paralysis	5 (1)	(<5)	5 (1)	0 (0)	(<5)	12 (1)
Other neurological disorders	22 (4)	22 (3)	18 (4)	14 (7)	21 (7)	60 (7)
Chronic obstructive pulmonary disease	78 (15)	99 (15)	107 (22)	42 (21)	77 (24)	252 (28)
Diabetes, uncomplicated	169 (32)	229 (34)	201 (41)	88 (44)	141 (45)	409 (46)
Diabetes, complicated	101 (19)	159 (24)	135 (27)	58 (29)	95 (30)	305 (34)
Hypothyroidism	56 (10)	94 (14)	87 (18)	41 (20)	54 (17)	196 (22)
Renal failure	23 (4)	34 (5)	47 (10)	28 (14)	53 (17)	154 (17)
Liver disease	336 (63)	433 (64)	324 (66)	108 (54)	189 (60)	563 (63)
Peptic ulcer disease, excluding bleeding	43 (8)	42 (6)	32 (7)	15 (8)	26 (8)	60 (7)
AIDS/HIV	0 (0)	(<5)	(<5)	0 (0)	0 (0)	(<5)
Lymphoma	5 (1)	11 (2)	12 (2)	(<5)	(<5)	12 (1)
Rheumatoid arthritis/collagen vascular disease	14 (3)	24 (4)	18 (4)	12 (6)	23 (7)	52 (6)
Coagulopathy	30 (6)	55 (8)	54 (11)	21 (10)	27 (9)	115 (13)
Obesity	112 (21)	155 (23)	110 (22)	58 (29)	76 (24)	202 (23)
Weight loss	208 (39)	271 (40)	208 (42)	80 (40)	137 (43)	402 (45)
Fluid and electrolyte disorders	136 (25)	205 (31)	179 (36)	63 (31)	110 (35)	317 (36)
Blood loss anemia	8 (2)	15 (2)	10 (2)	(<5)	8 (3)	24 (3)
Deficiency anemia	41 (8)	56 (8)	49 (10)	20 (10)	43 (14)	108 (12)
Alcohol abuse	38 (7)	38 (6)	30 (6)	6 (3)	7 (2)	42 (5)
Drug abuse	18 (3)	25 (4)	14 (3)	8 (4)	10 (3)	34 (4)
Psychosis	(<5)	0 (0)	(<5)	0 (0)	(<5)	8 (1)
Depression	86 (16)	122 (18)	94 (19)	47 (23)	71 (22)	172 (19)

Abbreviations: FFX, FOLFIRINOX (fluorouracil, leucovorin, irinotecan, and oxaliplatin); GnP, gemcitabine with nab-paclitaxel; mFFX, modified FOLFIRINOX.

Table S3. Healthcare Costs Among Commercial and Medicare Advantage Insurance Patient Groups Treated With 1L FFX, mFFX, or GnP

	**Commercial**			**Medicare Advantage**		
	**FFX**	**mFFX**	**GnP**	**FFX**	**mFFX**	**GnP**
**Total patients, n**	536	673	494	201	317	894
**Costs during 1L therapy, 2023 USD**						
TCoC						
Mean (SD)	137 813 (127 504)	120 109 (112 208)	133 042 (154 248)	110 788 (98 492)	98 667 (83 437)	110 211 (100 150)
Median (IQR)	102 719 (60 370-170 786)	92 086 (45 728-156 954)	96 889 (56 220-160 087)	84 760 (45 919-140 804)	72 931 (37 502-136 483)	86 354 (47 597-142 861)
Range	4940, 1 168 459	4815, 1 055 378	7704, 2 168 794	4889, 772 024	2456, 563 368	4419, 1 283 060
Inpatient						
Mean (SD)	19 039 (35 471)	15 809 (33 733)	20 158 (42 729)	17 043 (29 173)	16 376 (31 804)	19 789 (45 094)
Median (IQR)	0 (0-25 784)	0 (0-17 280)	0 (0-23 775)	0 (0-24 997)	0 (0-23 020)	0 (0-26 368)
Range	0, 253 774	0, 259 641	0, 428 649	0, 174 761	0, 191 677	0, 800 239
ICU						
Mean (SD)	1061 (7293)	691 (6969)	830 (5139)	1034 (7048)	892 (4712)	1732 (9021)
Median (IQR)	0 (0-0)	0 (0-0)	0 (0-0)	0 (0-0)	0 (0-0)	0 (0-0)
Range	0, 120 241	0, 156 072	0, 87 634	0, 89 033	0, 48 051	0, 120 241
N omitted	0	0	0	0	0	<5
Non-ICU						
Mean (SD)	17 978 (33 731)	15 118 (32 291)	19 327 (41 795)	16 009 (27 977)	15 484 (30 769)	18 071 (41 770)
Median (IQR)	0 (0-24 572)	0 (0-16 094)	0 (0-22 375)	0 (0-23 998)	0 (0-19 717)	0 (0-24 459)
Range	0, 253 419	0, 254 070	0, 419 711	0, 174 081	0, 191 677	0, 711 392
Outpatient						
Mean (SD)	118 774 (121 265)	104 300 (104 318)	112 884 (142 080)	93 744 (95 444)	82 291 (76 170)	90 422 (81 371)
Median (IQR)	83 006 (46 515-148 755)	75 189 (38 512-136 171)	78 438 (42 531-136 608)	64 501 (34 597-117 160)	58 131 (27 068-113 956)	68 702 (34 856-118 853)
Range	4940, 1 151 127	3628, 1 055 378	5041, 2 106 578	3464, 772 024	2456, 520 706	2408, 767 556
Chemotherapy drug						
Mean (SD)	10 916 (21 647)	7 653 (10 054)	60 466 (112 589)	8 028 (11 044)	6 016 (7 688)	49 263 (49 373)
Median (IQR)	6245 (3037-11 323)	5048 (2344-8655)	38 497 (18 462-70 425)	4928 (2121-9351)	3894 (1683-7346)	35 394 (15 170-64 686)
Range	143, 401 585	20, 104 025	3446, 2 074 974	250, 91 963	270, 79 444	907, 548 670
N omitted	0	0	0	<5	0	<5
Chemotherapy administration						
Mean (SD)	25 458 (33 350)	22 795 (24 309)	12 206 (15 766)	25 512 (36 352)	21 524 (22 317)	11 103 (13 089)
Median (IQR)	17 386 (9431-30 463)	16 084 (8018-29 925)	7956 (3598-14 454)	17 440 (7270-30 147)	13 541 (6189-28 874)	6924 (3070-13 124)
Range	292, 403 790	928, 293 186	328, 171 978	557, 413 930	0, 162 937	0, 89 580

Table S3. Healthcare Costs Among Commercial and Medicare Advantage Insurance Patient Groups Treated With 1L FFX, mFFX, or GnP

	**Commercial**			**Medicare Advantage**		
	**FFX**	**mFFX**	**GnP**	**FFX**	**mFFX**	**GnP**
G-CSF						
Mean (SD)	38 074 (56 593)	27 823 (41 166)	4 029 (14 181)	30 535 (56 630)	24 596 (39 286)	2412 (9115)
Median (IQR)	19 421 (675-50 796)	11 553 (0-41 652)	0 (0-0)	12 699 (501-40 232)	8 220 (0-38 545)	0 (0-0)
Range	0, 593 415	0, 305 528	0, 129 259	0, 622 081	0, 352 524	0, 99 882
Radiation therapy						
Mean (SD)	4044 (18 314)	4022 (17 477)	974 (8165)	2438 (12 016)	3330 (13 893)	1018 (7548)
Median (IQR)	0 (0-0)	0 (0-0)	0 (0-0)	0 (0-0)	0 (0-0)	0 (0-0)
Range	0, 211 235	0, 170 917	0, 151 593	0, 123 811	0, 112 562	0, 138 496
Other Rx						
Mean (SD)	7497 (16 229)	9267 (42 115)	5055 (9081)	3493 (5272)	4412 (7723)	3436 (8051)
Median (IQR)	2431 (675-8073)	2728 (574-7586)	1839 (321-5805)	1123 (143-4530)	1350 (164-5060)	953 (133-3720)
Range	0, 229 920	0, 917 729	0, 83 206	0, 28 253	0, 56 235	0, 123 340
Other outpatient						
Mean (SD)	32 784 (33 774)	32 883 (34 845)	30 232 (37 391)	23 792 (18 801)	22 414 (20 239)	23 274 (29 539)
Median (IQR)	23 753 (13 850-40 256)	23 751 (13 583-39 963)	20 078 (10 924-35 325)	19 208 (9530-31 459)	16 651 (8773-28 248)	17 142 (8381-29 141)
Range	131, 332 588	534, 423 838	219, 343 389	606, 99 029	469, 149 484	39, 563 867
N omitted	0	<5	<5	0	0	<5
Follow-up time, months						
Mean (SD)	5.8 (5.3)	5.3 (4.3)	4.8 (4.3)	5.1 (4.1)	4.5 (3.5)	4.3 (3.3)
Median (IQR)	4.6 (2.3-7.4)	4.6 (2.1-6.9)	3.7 (2.1-6.2)	3.9 (2.1-7.2)	3.5 (1.9-6.2)	3.3 (1.9-6.0)
Range	0.4, 62.7	0.6, 32.0	0.5, 54.3	0.9, 23.2	1.0, 22.2	0.5, 20.9

Abbreviations: 1L, first line; admin, administration; chemo, chemotherapy; FFX, FOLFIRINOX (fluorouracil, leucovorin, irinotecan, and oxaliplatin); GnP, gemcitabine with nab-paclitaxel; IQR, interquartile range; mFFX, modified FOLFIRINOX; Rx, prescription; USD, US dollars.

Table S4. One-Way ANOVA and Games-Howell Test Statistics for Mean Cost Outcomes

Cost Outcome	Comparison	ANOVA *P*	Difference in Means	95% CI	Pairwise *P*
TCoC across all regimens and payer types	All regimens	<.001			
	FFX, Com vs MA		−27 025	−52 395, −1655	<.001
	mFFX, Com vs MA		−21 442	−39 659, −3225	<.001
	GnP, Com vs MA		−22 831	−44 850, −812	<.001
	Com FFX vs mFFX		−17 704	−37 696, 2288	.005
	Com FFX vs GnP		−4771	−30 070, 20 528	.974
	Com mFFX vs GnP		12 933	−10 423, 36 289	.222
	MA FFX vs mFFX		−12 121	−36 126, 11 884	.319
	MA FFX vs GnP		−577	−22 698, 21 544	.999
	MA mFFX vs GnP		11 544	−4919, 28 007	.053
Commercial group					
Inpatient	All regimens	.106			
ICU	All regimens	.670			
Non-ICU	All regimens	.119			
Outpatient	All regimens	.082			
Chemotherapy drug	All regimens	<.001			
	FFX vs mFFX		−3263	−5640, −886	<.001
	FFX vs GnP		49 550	37 443, 61 657	<.001
	mFFX vs GnP		52 813	40 870, 64 756	<.001
Chemotherapy admininstration	All regimens	<.001			
	FFX vs mFFX		−2663	−6697, 1371	.073
	FFX vs GnP		−13 252	−17 022, −9482	<.001
	mFFX vs GnP		−10 589	−13 347, −7831	<.001
G-CSF	All regimens	<.001			
	FFX vs mFFX		−10 251	−17 092, −3410	<.001
	FFX vs GnP		−34 045	−39 981, −28 109	<.001
	mFFX vs GnP		−23 794	−27 809, −19 779	<.001
Radiotherapy	All regimens	<.001			
	FFX vs mFFX		−22	−2460, 2416	.999
	FFX vs GnP		−3070	−5118, −1022	<.001
	mFFX vs GnP		−3048	−4849, −1247	<.001
Other OP Rx	All regimens	.001			
	FFX vs mFFX		1770	−2381, 5921	.333
	FFX vs GnP		−2442	−4347, −537	<.001
	mFFX vs GnP		−4212	−8143, −281	.001
Other OP medical	All regimens	.410			
Medicare Advantage group					
Inpatient	All regimens	.295			
ICU	All regimens	.104			
Non-ICU	All regimens	.457			
Outpatient	All regimens	.203			
Chemotherapy drug	All regimens	<.001			
	FFX vs mFFX		−2012	−4109, 85	.004
	FFX vs GnP		41 235	36 950, 45 520	<.001
	mFFX vs GnP		43 247	39 241, 47 253	<.001

Table S4. One-Way ANOVA and Games-Howell Test Statistics for Mean Cost Outcomes

Cost Outcome	Comparison	ANOVA *P*	Difference in Means	95% CI	Pairwise *P*
Chemotherapy admininstration	All regimens	<.001			
	FFX vs mFFX		−3988	−10 711, 2735	.120
	FFX vs GnP		−14 409	−20 549, −8269	<.001
	mFFX vs GnP		−10 421	−13 544, −7298	<.001
G-CSF	All regimens	<.001			
	FFX vs mFFX		−5939	−16 684, 4806	.158
	FFX vs GnP		−28 123	−37 582, −18 664	<.001
	mFFX vs GnP		−22 184	−27 428, −6940	<.001
Radiotherapy	All regimens	.008			
	FFX vs mFFX		892	−1817, 3601	.518
	FFX vs GnP		−1420	−3506, 666	.062
	mFFX vs GnP		−2312	−4242, −382	<.001
Other OP Rx	All regimens	.144			
Other OP medical	All regimens	.717			

Abbreviations: CI, confidence interval; Com, commercial; MA, Medicare Advantage; OP, outpatient; Rx, prescription.

Table S5. Chemotherapy Administration Average Cost per HCPCS/CPT Code Among Patients Treated With 1L FFX, mFFX, and GnP

HCPCS/ CPT Code	Description	Average Cost per Patient, USD			Difference	
		FFX (n = 464)	mFFX (n = 532)	GnP (n = 888)	FFX vs GnP	mFFX vs GnP
96416	Chemotherapy prolonged infusion with pump	2117	2103	2	2115	2101
96411	Chemotherapy IV push additional drug	1293	116	41	1252	75
96415	Chemotherapy IV infusion additional hr	1283	1530	33	1250	1497
J1453	Fosaprepitant injection	1343	1356	218	1125	1139
96368	Therapeutic/diagnostic concurrent infusion	1060	984	24	1036	960
J0185	Aprepitant injection, 1 mg	1010	1239	104	906	1135
96372	Therapeutic/prophylactic/diagnostic injection subcutaneous/intramuscular	937	863	130	807	733
96367	Therapeutic/prophylactic/diagnostic additional sequence IV infusion	1321	1237	529	792	708
96375	Therapeutic/prophylactic/diagnostic injection new drug add-on	1916	1820	1188	728	632
G0498	Chemotherapy extend IV infusion with pump	441	576	0	441	576
J2469	Palonosetron hydrochloride	1274	1125	880	394	245
96360	Hydration IV infusion initial	526	490	168	358	322
96361	Hydration IV infusion add-on	487	428	184	303	244
J7060	5% dextrose/water	164	160	2	162	158
96366	Therapeutic/prophylactic/diagnostic IV infusion add-on	195	148	34	161	114
96365	Therapeutic/prophylactic/diagnostic IV infusion initial	236	340	88	148	252
96374	Therapeutic/prophylactic/diagnostic injection IV push	223	363	119	104	244
96521	Refill/maintenance of a portable pump	101	120	0	101	120
J0881	Darbepoetin alfa, non-end-stage renal disease	44	84	209	-165	-125
96417	Chemotherapy IV infusion each additional sequence	1447	1408	1718	-271	-310
96413	Chemotherapy IV infusion 1 hr	2787	2776	3690	-903	-914

Abbreviations: CPT, Current Procedural Terminology; HCPCS, Healthcare Common Procedure Coding System; FFX, FOLFIRINOX (5-fluorouracil, leucovorin, irinotecan, and oxaliplatin); GnP, gemcitabine with nab-paclitaxel; mFFX, modified FOLFIRINOX; USD US dollars.
